# Preserving subject variability in group fMRI analysis: performance evaluation of GICA vs. IVA

**DOI:** 10.3389/fnsys.2014.00106

**Published:** 2014-06-26

**Authors:** Andrew M. Michael, Mathew Anderson, Robyn L. Miller, Tülay Adalı, Vince D. Calhoun

**Affiliations:** ^1^Autism and Developmental Medicine Institute, Geisinger Health SystemDanville, PA, USA; ^2^The Mind Research Network and LBERIAlbuquerque, NM, USA; ^3^Department of Computer Science and Electrical Engineering, University of MarylandBaltimore, MD, USA; ^4^Department of Electrical and Computer Engineering, University of New MexicoAlbuquerque, NM, USA

**Keywords:** fMRI, group ICA method, brain network analysis, functional connectivity, independent vector analysis

## Abstract

Independent component analysis (ICA) is a widely applied technique to derive functionally connected brain networks from fMRI data. Group ICA (GICA) and Independent Vector Analysis (IVA) are extensions of ICA that enable users to perform group fMRI analyses; however a full comparison of the performance limits of GICA and IVA has not been investigated. Recent interest in resting state fMRI data with potentially higher degree of subject variability makes the evaluation of the above techniques important. In this paper we compare component estimation accuracies of GICA and an improved version of IVA using simulated fMRI datasets. We systematically change the degree of inter-subject spatial variability of components and evaluate estimation accuracy over all spatial maps (SMs) and time courses (TCs) of the decomposition. Our results indicate the following: (1) at low levels of SM variability or when just one SM is varied, both GICA and IVA perform well, (2) at higher levels of SM variability or when more than one SMs are varied, IVA continues to perform well but GICA yields SM estimates that are composites of other SMs with errors in TCs, (3) both GICA and IVA remove spatial correlations of overlapping SMs and introduce artificial correlations in their TCs, (4) if number of SMs is over estimated, IVA continues to perform well but GICA introduces artifacts in the varying and extra SMs with artificial correlations in the TCs of extra components, and (5) in the absence or presence of SMs unique to one subject, GICA produces errors in TCs and IVA estimates are accurate. In summary, our simulation experiments (both simplistic and realistic) and our holistic analyses approach indicate that IVA produces results that are closer to ground truth and thereby better preserves subject variability. The improved version of IVA is now packaged into the GIFT toolbox (http://mialab.mrn.org/software/gift).

## Introduction

Investigating macro-level brain circuitry using functional magnetic resonance imaging (fMRI) is of great interest to the neuroimaging community. Functional brain connectivity maps are widely used to investigate healthy and diseased populations in order to identify aberrant networks patterns (Garrity et al., 2007; Greicius, 2008; Bassett and Bullmore, 2009). Recently there has been increased interest to identify brain networks of resting state fMRI (rsMRI); data acquired while a subject is not performing a particular task. Approaches used to analyze resting state data include seed-based correlation analyses (Cohen et al., 2008) and data-driven approaches such as independent component analysis (ICA) (McKeown et al., 1998). ICA can identify multiple coherent networks without the need for an *a priori* seed voxel, region of interest or model timecourse. Group ICA (GICA), a framework that includes ICA, is widely applied to group fMRI data (Calhoun et al., 2001; Calhoun and Adali, 2012). GICA provides a solution to the problem of permutation ambiguity of ICA by matching components across subjects; first estimating the group level components and then estimating single subject spatial maps (SM) and time courses (TC). GICA makes no assumption about the temporal consistency across subjects but does assume spatial stationarity. It can capture inter-subject spatial variability, but there are limits (Allen et al., 2012). Independent vector analysis (IVA), a multivariate extension of ICA, was introduced by Lee et al. as an alternate way of performing group fMRI analyses while avoiding the permutation ambiguity of ICA (Lee et al., 2007, 2008). Lee et al. indicated through their simplistic experiments that IVA can better capture subject variability; however, a full comparison of the limits of both GICA and IVA was not performed. In this paper, we compare component estimation accuracies of GICA and IVA using simulated fMRI data under varying types and degrees of inter-subject spatial variability. In addition we find several important characteristics of both methods and make recommendations to users. FMRI data were simulated using SimTB (http://mialab.mrn.org/software/), a recently developed toolbox that generates data under the spatio-temporal assumption of ICA and IVA (Erhardt et al., 2012).

Changes in brain morphology between subjects and even within the same subject over time are well reported (Giedd et al., 1999). Variability among subjects in functional brain network patterns (distinct from anatomic patterns) was recently shown in Khullar et al. (2011). Spatial and temporal differences across subjects can exist in fMRI networks especially in resting state studies where subjects do not follow an assigned task. Effectively preserving subject specific activation patterns is critical to identify differences and potential biomarkers in patient populations. Subject variability in functional activity can occur in the amplitude of activation, spatial location/extent of activation and temporal variations. In the ICA/IVA domain, subject variability can be measured in terms of variations in the SMs and TCs.

ICA can be successfully applied to separate statistically independent SMs from fMRI data. ICA is particularly useful since *a priori* knowledge of these sources is not required. In the application of ICA to fMRI data, ICA assumes that the fMRI data is a linear mixture of SMs and TCs and decomposes the fMRI data to find temporally coherent SMs that are spatially independent (statistically). Application of ICA to individual subject fMRI data is relatively straightforward. FMRI data is separated into a user specified number of SMs and their corresponding TCs. When ICA is applied separately to multiple subjects on an individual basis, comparing SMs across subjects to make group level inferences becomes challenging due to inter-subject spatial variability of SMs. With such spatial variability, especially when the number of SMs is high, it is not easy to cluster similar SMs across subjects to perform statistical analyses at the group level. GICA provides a way to address this problem by estimating a decomposition from all subjects' data (Calhoun et al., 2001). In GICA subject data are first temporally concatenated, followed by a group level PCA reduction, and then application of ICA yielding group level components. Finally, a back-reconstruction step is applied to make subject specific SMs and TCs (Erhardt et al., 2011). The GICA framework has been applied extensively across many studies, in both healthy and patient populations, to make inferences about intrinsic networks (Sorg et al., 2007; Calhoun et al., 2009; Allen et al., 2011).

IVA was first introduced as a blind source separation technique to separate time delayed and convolved signals using higher order frequency dependencies. IVA uses a multivariate extension of the mutual information cost function used in ICA (Lee et al., 2007). In the original IVA application the source signal was made independent within each frequency bin while enforcing higher order dependencies across frequency bins. In a previous study IVA was applied to multi-subject fMRI data to construct individual SMs and TCs (Lee et al., 2008). In GICA the input matrix is the PCA reduced two dimensional group matrix, in contrast, in IVA subject data are not mixed together but kept separate along the 3rd dimension of the input matrix. IVA maximizes an objective function that considers both the independence of within subject SMs and the dependence of similar SMs across subjects. With this strategy the back reconstruction step needed in GICA to estimate subject specific SMs is avoided. Further, since IVA accounts for the dependence of similar components, component ordering across subjects is preserved making group analyses across subjects straightforward.

Studies have investigated the performance of GICA (Allen et al., 2012) and IVA (Lee et al., 2008). In Lee et al. (2008) a two trial based simulated dataset was used to test the performance of ICA and IVA under slight inter-subject variability and noise levels. Results of their experiment showed that, compared to GICA, IVA captured inter-subject variability better. Most of their results compare estimations from real fMRI data using GLM, GICA, and IVA. Although marginal variability to subject TCs and SMs were added to the task-related data, they did not evaluate the performance of IVA at high variability of SMs and TCs. Resting state fMRI data do not follow a task like TC and its activation patterns can have significant inter-subject variability.

Recent improvements (IVA-GL) have been made to the IVA algorithm to achieve reliable source separation for linearly dependent Gaussian and non-Gaussian sources and extend the application of IVA to separate sources with linear dependence (Anderson et al., 2010). In Dea et al. (2011) realistic fMRI datasets were simulated using SimTB to investigate the performance of two different IVA approaches, IVA-GL and IVA-GJD (Li et al., 2011). In Ma et al. (2013) it was shown that performance of IVA in capturing group difference improved as group variability increased and that GICA performed better at low variability. Using mutual information as a metric, they showed that the IVA algorithm outperforms GICA in capturing spatial inter-subject variability. The initial results of the above studies provide evidence that IVA can provide improved component estimations in datasets where there is SM variability. However, these studies did not evaluate the performance of GICA and IVA under different degrees of subject variability and other estimation parameters.

In this paper, we compare SM and TC estimation accuracies of GICA vs. IVA under spatial variation of SMs between subjects. In addition to comparing estimation accuracies of the component that is varied between subjects, we also inspect changes in all other components of the decomposition. In other words, we investigate all elements of the cross correlation matrices between the ground truth components (GND) and reconstructed or estimated components (EST) of all SMs and TCs. In our initial experiments (*Experiments 1–3*), we select a lower number of subjects and components to make result presentation easier. In *Experiment 4* we repeat with a larger number of subjects and components. We simulate several scenarios of inter-subject variability; *Experiment 1:* SM amplitude at different noise levels, *Experiment 2:* different types of spatial variability (translation, rotation, and size) in one SM, *Experiment 3:* combinations of different types of variability in two SMs, *Experiment 4:* all SMs in all subjects have a combination of spatial variability. Under each experiment, we perform several sub–experiments to address effects of different degrees of variability, slight spatial overlap of SMs, effects of overestimation of component, effects of presence or absence of components, effects of different noise levels and other variations.

## Methods

### GICA vs. IVA

Main steps of GICA and IVA while applying these techniques to perform group analysis of fMRI data are briefly presented in this section.

Definitions of main notations used:

*M*: Total number of subjects in the group

*V*: Total number of in-brain voxels, *V* is common to all *M* subjects

*T*: Number of time points in the fMRI data, *T* is common to all *M* subjects

*C*: Number of spatially independent components (user defined parameter), *C* is common to all *M* subjects

**Y**_*i*_ [*T* × *V*]: fMRI data matrix from the *i*^*th*^ subject, *i* = 1 to *M*. **Y**_*i*_ is formed after the three dimensional brain voxels are stacked along the columns of **Y**_*i*_ and time points along the rows

**X**: Input matrix to ICA/IVA algorithm. For ICA dimensions of **X** are [*C* × *V*], for IVA dimensions of **X** are [*C* × *V* × *M*].

**Ṡ**_*i*_ [*C* × *V*]: Contains the *i*^*th*^ subject's *C* independent spatial maps (SMs) estimated by the algorithm

**Ṙ**_*i*_ [*T* × *C*]: Contains the *i*^*th*^ subject's *C* independent timecourses (TCs) corresponding to the SMs

**Figure 1:**
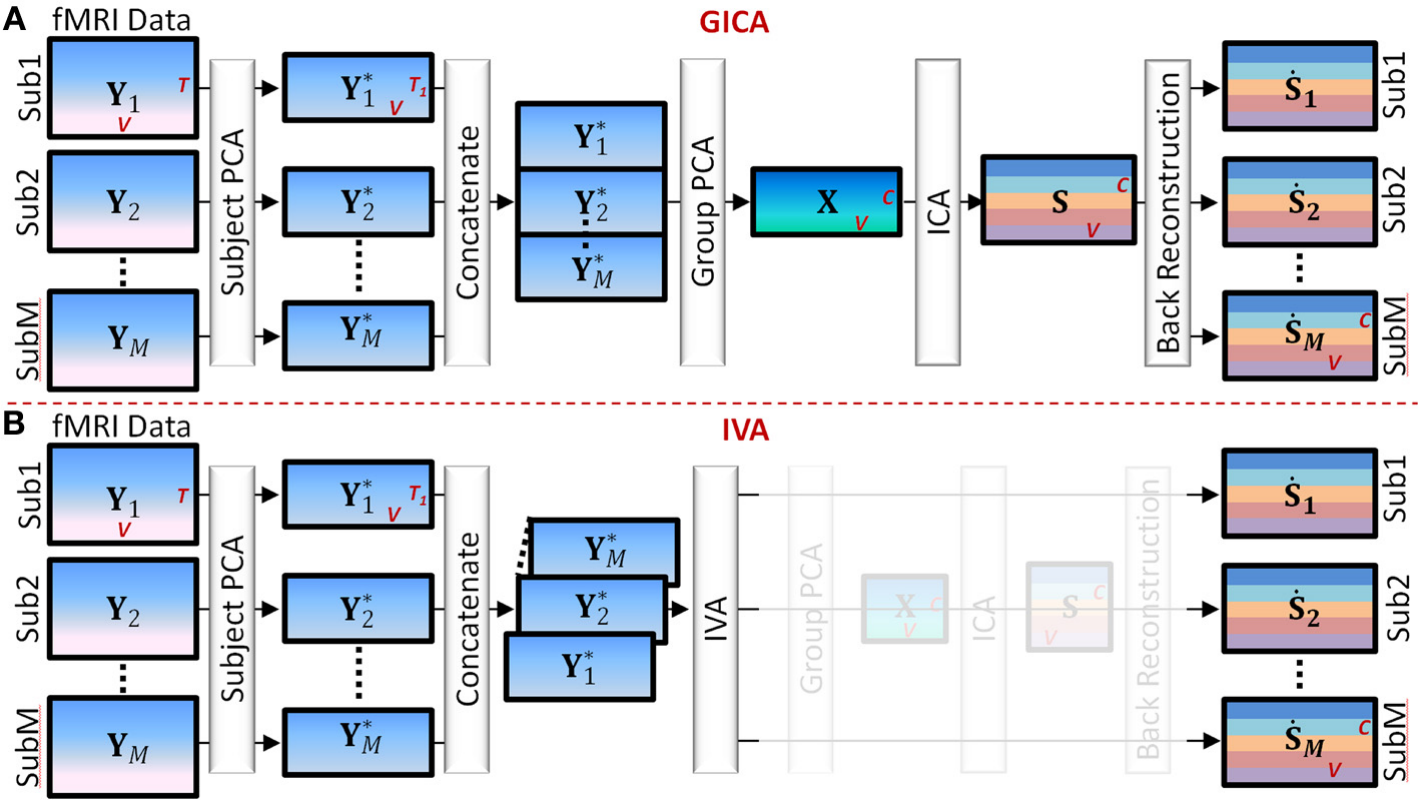
**Main Steps of GICA and IVA (see Section GICA vs. IVA for more details). (A)** GICA: (i) subject level principal component analysis (PCA) on each subject's fMRI data (**Y**_**i**_) of size time points (*T*) by voxels (*V*) results in matrices (Y^*^_i_) of size *T*_1_ by *V*, *T*_1_ is a user specified number, (ii) concatenate Y^*^_i_ along the time domain, (iii) apply group PCA on the concatenated group matrix to get matrix **X** of size *C* by *V*, where *C* is a user specified number, (iv) apply independent component analysis (ICA) on **X** to obtain group level spatial maps (SMs) and time courses (TCs) and (v) apply a back reconstruction method to obtain subject specific SMs and TCs. **(B)** IVA: (i) same as GICA, (ii) concatenate Y^*^_i_ along the 3rd dimension keeping each subject's data separate, (iii) apply IVA to obtain subject specific SMs and TCs.

Main steps of Group GICA (see Figure 1).

1. *Subject level principal component analysis (PCA):* Each subject's data matrix (**Y**_*i*_) [*T* × *V*] is reduced along the time domain to retain *T*_1_ principal components, where *T*_1_ < *T*. Let Y^*^_i_ [*T*_1_ × *V*] be the subject level PCA reduced matrix of the *i*^*th*^ subject.
2. *Temporal concatenation of subject data:* PCA reduced subject matrices (Y^*^_i_) are concatenated along the temporal domain (rows) to form the group fMRI matrix Y^*^ = [Y^*T^_1_, Y^*T^_2_, … Y^*T^_*M*_]^*T*^ of size [*MT*_1_ × *V*].
3. *Group level PCA:* Perform PCA reduction on the Y^*^ matrix and retain *C* principal components. Let **X** [*C* × *V*] be the group level PCA reduced matrix.
4. *Independent Component Analysis (ICA):* Perform spatial ICA on the **X** matrix to decompose **X** into group level SMs given by **X** = **AS**, where **A** [*C* × *C*] is related to component TCs and **S** [*C* × *V*] contains group level SMs.
5. *Subject level back-reconstruction:* There are several methods to back-project group level maps to subject data to obtain subject specific SMs (**Ṡ**_*i*_) and TCs (**Ṙ**_*i*_), as described in Erhardt et al. (2011) for a detailed explanation of the different techniques. In this work we used the spatio-temporal regression (STR) method to back-reconstruct subject specific SMs and TCs. **Ṙ**_*i*_ is given by **Ṙ**_*i*_ = **Y**_*i*_**S**^−^, and **Ṡ**_*i*_ = **Ṙ**^−^_*i*_**Y**_*i*_ where **S**^−^ and **Ṙ**^−^_*i*_ are the pseudo-inverses of **S** and **Ṙ**_*i*_, respectively.

Main steps of IVA (see Figure 1).

1. *Subject level PCA*: As in GICA, each subject's data matrix (**Y**_*i*_) [*T* × *V*] is reduced in the time domain to compute Y^*^_i_ [*C* × *V*], where *C* is the number of desired components.
2. *Concatenation of subject data*: Unlike in GICA, in IVA subject data are concatenated along the third dimension to form the **X** matrix of dimension [*C* × *V* × *M*].
3. *Independent Vector Analysis (IVA)*: Perform IVA on the **X** matrix. In IVA, the decomposition is performed on the three-dimensional **X** matrix while keeping each subject's SMs and mixing matrices unmixed between subjects. The decomposition yields three dimensional matrices given by **X**_*i*_ = **A**_*i*_**S**_*i*_, where *i* = 1 to *M* and denotes the *i*^*th*^ subject (in third dimension).
4. *Subject Maps and time courses:* In IVA reconstruction of subject level SMs and TCs are straightforward as each subject's data is in its own space stacked along the third dimension of the matrices. Subject specific spatial map **Ṡ**_*i*_ is given by **Ṡ**_*i*_ = **A**^−1^_*i*_**X**_*i*_ and the subject specific timecourse is given by **Ṙ**_*i*_ = **Y**_*i*_**Ṡ**^−^_*i*_.

### Simulation setup

We used SimTB (Erhardt et al., 2012), a MATLAB toolbox available at (http://mialab.mrn.org/software/simtb/index.html), to simulate fMRI datasets. SimTB was designed to generate fMRI datasets under the assumption of spatiotemporal separability of the fMRI data. In other words it is assumed that the fMRI data can be given as a product of spatial and temporal processes (product of SMs and TCs). In SimTB the user defines the number of SMs and selects them from a predesigned set. The TCs of SMs were generated from a zero mean unit variance normal distribution. To create different types of fMRI datasets we changed the following: SM amplitude (or percentage signal change), baseline intensity, noise, spatial variability of SMs (translation, rotation, size) and varied TCs for each component and subject. The SMs were represented as 2D axial images of size 100 × 100 voxels. We reduced the number of voxels in the SMs (compared to real fMRI) to increase the speed of dataset generation, GICA/IVA decomposition and to reduce the hard disk space needed as a very large number of fMRI datasets were simulated in this study.

In order to better grasp the functionalities of the algorithms, for *Experiments 1–3*, we use a lower number of subjects and components with the following simulation parameters and for *Experiment 4* we use a larger number of subjects and components (*Experiment 4* numbers are presented below within parenthesis).

Number of subjects in the group, *M* = 5 (20)

Size of image slice = 100 × 100 voxels, number of in-brain voxels (*V*) = 7688

Number of time points, *T* = 150

Number of SMs in the data, *C* = 6 (15)

GND: Ground truth component

EST: Estimated or reconstructed component

Subi: *ith* Subject

SMj: *jth* SM

TCj: *jth* TC

Main Steps of data simulation, component estimation, and result comparison:

1. *Simulate fMRI dataset:* In addition to the above parameters the following can be changed in SimTB: SM sources and their presence in subjects, SM translation in voxels, SM rotation in degrees, SM spread (size), baseline signal intensity, SM signal amplitude, and contrast to noise ratio (CNR). SimTB simulates fMRI datasets with the following main steps, for a detailed description of each of these steps refer to Erhardt et al. (2012)
  - TC *generation*: in our experiments, all TCs are generated as a random time series with an additional constraint of near zero correlation between the TCs of a subject. With this constraint we allow maximum variability in the time domain to make the evaluation of spatial variability as the main focus of our project.
  - SM *generation*: SMs are generated as activation blobs defined by 2D Gaussian distributions with varying spatial characteristics (translations, rotations, size etc).
  - *Make baseline intensity*: for each subject a variable baseline intensity map is computed, voxels outside the brain mask are set to zero.
  - *Scale* SMs: SMs are scaled according to the percentage signal changes. Percentage signal changes are defined as the peak-to-peak signal change relative to the baseline. Varying percentage signal change values can be assigned to each component and subject.
  - *Mix* SMs *with* TCs: SMs and TCs are linearly combined as the matrix product and then each subject's baseline frame is added.
  - *Add noise*: Rician noise is added to the data according to the CNR values assigned to each subject.
2. *GICA/IVA Decomposition*: Simulated fMRI datasets of the *M* subjects are fed into the GICA and IVA algorithms separately to reconstruct SMs and TCs. We briefly describe the steps involved in the decomposition and the parameters used for estimation.
  - *Subject level PCA*: In this project, the actual number of components in the dataset are known and since the main focus is on analyzing the impact of subject variability, we reduce each subject's data to the actual number of components in the simulated data set, that is *T*_1_ = *C*.
  - *Group level PCA for ICA:* Here again we reduce the temporally concatenated group dataset to *C* number of principal components (except *Experiments 2h and 4c* where we perform overestimation). In real applications there are ways to estimate the number of components using methods such as minimum description length (Wax and Kailath, 1985; Li et al., 2007).
  - *Component Estimation:* For ICA we feed the two dimensional **X** matrix (group level PCA reduced whitened matrix) to the *Infomax* algorithm (Bell and Sejnowski, 1995). For IVA we first feed in the three dimensional **X** matrix (subject level PCA reduced whitened matrix) to the second order IVA-GL algorithm (Anderson et al., 2010) to obtain a set of unmixing matrices. We re-run the data with the original IVA algorithm (Lee et al., 2007) where we initialize the unmixing matrix with the results obtained from IVA-GL. Default values set by the original GICA and IVA designers were used for parameters such as learning rate, maximum number of iterations and terminations threshold.
3. *Component Scaling and Sorting*: In GICA and IVA decompositions have scaling and sign ambiguity (the amplitude and sign of the SMs and TCs can be scaled provided that the product of the scaling factors is unity). The amplitude or the sign of the components do not convey useful information by themselves. The signs of each SM and the corresponding TC were flipped based on the skewness of the SM. If the skewness of the SM was less than zero the signs of both SM and TC were flipped (multiplied by negative one). In an effort to display all recovered SMs in a consistent manner, we scaled all the SMs to values between negative one and positive one. TCs were z-scored, to have zero mean and unit variance. Reconstructed components were matched with GND, based on the spatial cross correlation matrix between the GND and estimated (EST) SMs. Component pairing is made based on the descending order of the GND–EST correlations using a greedy algorithm where once an EST SM is paired with a GND SM, that SM is not selected again to pair up with another GND. The component sorting scheme used is an important step in the performance evaluation of the ICA/IVA algorithms. At higher subject variability, the EST SMs can be a mixture of GND SMs with significant correlations with more than one GND SM.

### Simulation experiment setup

**Figure 2:**
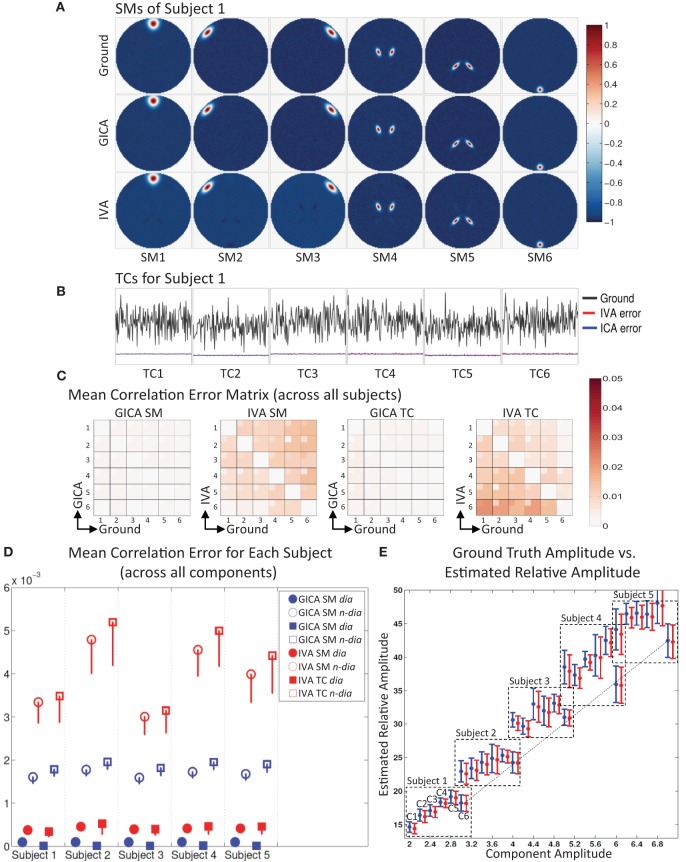
**Results of Experiment 1: Estimation accuracy with variation in only the Amplitude of SMs (no spatial variability of components). (A)** Spatial Maps (SM) of subject 1: 6 Ground SMs, GICA SM estimates, and IVA SM estimates are respectively presented in different rows. All SMs are scaled between −1 and +1. SMs of Subjects 2–5 are very similar to Subject 1. SMs show that both GICA and IVA perform very good estimations (IVA estimations had minor artifacts). **(B)** Time Courses (TC) of Subject 1. TCs are normalized to zero mean unit variance. Under each TC the error (shifted for better representation, but scale unchanged) between the estimates and ground are presented in blue for GICA and red for IVA. Compared to ground TC, the relative magnitudes of the errors are very small. **(C)** Mean correlation error matrix between ground and estimates is calculated for all subjects and the mean and standard deviation (mini-cell inside each cell) is presented for GICA and IVA and SM and TC separately (see “Definition of Result Evaluation Parameters” section for their definitions). All mean errors are less than 0.05. **(D)** Mean correlation errors across all components, presented for each subject separately. Mean errors are in blue for GICA and in red for IVA. Diagonal elements of the correlation error matrix are in filled shapes and non-diagonal in unfilled shapes. SMs are presented in circles and TCs in squares. All errors are in the order of 10^−3^ indicating that variability in SM amplitude does not affect component reconstructions. **(E)** Ground truth vs. estimated amplitude. Changes in SM amplitude can be recovered in a relative sense.

To isolate the effect of spatial overlap, in most of our experiments SMs were chosen such that even after adding spatial variability there was no spatial overlap between SMs. For *Experiments 1–3* we simulated data for 5 subjects with 6 components each (SMs and TCs for Sub1 is shown in Figure 2). For *Experiment 4* 20 subjects with 15 components each (SMs are presented in Supplementary Figure 1) were simulated.

#### Experiment 1 (change in amplitude)

The spatial locations of the components are kept constant across subjects, but the amplitude of the components is varied across subjects. This experiment was designed to test whether subject variability in component amplitude introduces errors in the estimated SMs and TCs. We also checked if variability in component amplitude (*Amp*_*GND*_) was preserved in the estimated components. The component amplitude of the estimated components (*Amp*_*EST*_) were calculated as the product of the standard deviation of the TC (σ_*TC*_) and the maximum intensity of SM (*max*_*SM*_), a metric introduced by Allen et al. (2012). *Amp*_*EST*_ calculations were made before we scaled the SMs between negative and positive one.

#### Experiment 2 (spatial change in one component)

In this experiment, we introduce different types of inter-subject spatial variability in just one of the six SMs. We check how estimation errors change as spatial variability increases.

#### Experiment 2a (vertical translation of SM1)

We translate SM1 (see Figure 2A) in the vertical direction between zero and Δ*d*_*max*_ number of voxels. SM1 of Sub1 is shifted by zero voxels (no translation), in Sub5 SM1 is shifted by Δ*d*_*max*_ voxels and in Sub2–Sub4 SM1 is shifted by Δ*d* voxels relative to the previous subject, where Δ*d* = Δ*d*_*max*_/*M*, *M* = 5 (number of subjects). We then repeat this test five times for incrementing values of Δ*d*_*max*_. The full width half max (FWHM) of SM1 is approximately equal to 10 voxels and Δ*d*_*max*_ was incremented as a multiple of the FWHM of SM1. In the first test Δ*d*_*max*_ was set to 5 voxels (or 0.5 FWHM) and for tests thereafter we incremented Δ*d*_*max*_ by 5 voxels.

#### Experiment 2b (spatial extent or size of SM2)

The size of SM1 was set to 0.1 for Sub1, Δ*s*_*max*_ for Sub5 and at increments of Δ*s* = Δ*s*_*max*_/*M* for Sub2–Sub4. This test was repeated five times at five different values of Δ*s*_*max*_, from 0.4 to 2.0 at intervals of 0.4. When Δ*s* is increased from 0.1 to 0.4 the size of SM2 is approximately doubled.

#### Experiment 2c: (Rotation of SM3)

In this sub-experiment we set the orientation of SM3 for Sub1 at 0°, Sub5 at Δθ_*max*_ and at increments of Δθ = Δθ_*max*_/*M* for Sub2–4. This test was repeated five times for five different values Δθ_*max*_ from 36 to 180° at intervals of 36°.

#### Experiment 2d (horizontal separation of SM5)

The horizontal separation between the two blobs of SM5 is varied. The FWHM of SM5 is approximately equal to 5 voxels and Δ*d*_*max*_ was incremented as a multiple of the FWHM of SM5 (from FWHM = 1 to 5).

#### Experiment 2e (component overlap)

The goal of this experiment is to check the effects of slight spatial overlap between SMs; this experiment is a continuation of *Experiment 2a*. Here we vertically translate SM1 across subjects. Here we set Δ*d*_*max*_ to 35 voxels. At this value of Δ*d*_*max*_, SM1 marginally overlaps with SM4 for Sub5 as shown in **Figure 4A**.

#### Experiment 2f (over estimation of model order)

In experiments 2a–2h the model order of the estimation was exactly matched with the actual number of components in the ground truth data. In this experiment, we test the effect of subject variability if the model order is over estimated. We repeat *Experiment 2a* (with Δ*d*_*max*_ = 25 voxels and *C* = 6 ground truth components) with 9 estimated components.

#### Experiment 2g (missing components)

We perform this experiment in two parts: (i) all 5 subjects have all 6 components except Sub1 where SM1 is not present. (ii) SM1 is present in Sub1 but absent in all other subjects.

#### Experiment 3 (spatial change in two components)

The purpose of this set of experiments was to check how spatial variation in two components changed estimation accuracy.

#### Experiment 3a (vertical translation of SM1 and size change of SM2)

This experiment is essentially several experiments of *Experiment 2b* nested within *Experiment 2a*.

#### Experiment 3b (vertical translation of SM1 and rotation of SM3)

This experiment is essentially several experiments of *Experiment 2c* nested within *Experiment 2a*.

#### Experiment 3c (vertical translation of SM1 and horizontal separation of SM5)

This experiment is essentially several experiments of *Experiment 2d* nested within *Experiment 2a*.

#### Experiment 4 (spatial variability in all components)

For this experiment we increased the number of subjects to *M* = 20 and the number of components to *C* = 15. We made this to roughly replicate the application of GICA or IVA to a real fMRI study. Here all components in all subjects undergo all forms of spatial variations as introduced in *Experiment 2.*

#### Experiment 4a (spatial variability with uniform distribution)

In this sub-experiment the degree of variability is picked from a uniform distribution, *U* (*a*, *b*), where *a* and *b* are varied depending on the type of spatial variability. For SM translations *a* = −2*k* and *b* = 2*k* (in voxels), for component rotation *a* = −10*k* and *b* = 10*k* (in degrees) and for component size *a* = 1 − 0.2*k* and *b* = 1 + 2*k*, where *k* is an integer from 1 to 5. A higher *k*-value corresponds to increased subject variability.

#### Experiment 4b (spatial variability with normal distribution)

Exact repetition of *Experiment 4a*, with degree of variability drawn from a normal distribution, *N* (μ, σ) where μ and σ are the mean and standard deviation of the distribution. The mean values in all variability distributions were kept at zero except for size where the mean value was maintained at one. At each value of *k* the standard deviation of the spatial variability for this experiment and *4a* were kept constant.

#### Experiment 4c (model order over estimation)

We repeat *Experiment 4a* applying spatial variability using *k* = 2 and at each iteration we increase the model order by 5 components from *C* = 15 (actual number of components) to *C* = 35.

#### Experiment 4d (estimation at high noise level)

We repeat Experiment 4a for a range of *CNRs*; *CNR* = 0.5 to *CNR* = 0.1 at *k* = 2.

### Definition of result evaluation parameters

We use correlation as the primary metric to compare similarity between GND and EST. Let **R**^**G**^_*i*_, **R**^**C**^_*i*_, **R**^**V**^_*i*_ of size [*C* × *C*] be the GND–GND, GND–GICA, and GND–IVA correlation matrices respectively for the *ith* subject. There will be two types of correlation matrices: one from SM and the other from TC. Let *r*^*C*^_*i*,*l*,*m*_ be the correlation between the *lth* GND component and *mth* GICA component for the *ith* subject. Let *r*^*V*^_*i*,*l*,*m*_ be the same for IVA. We define the correlation error matrices as the absolute value of the difference between the GND–GND correlations and GND–EST correlations. For GICA let **E**^**C**^_*i*_ = *abs* (**R**^**G**^_*i*_ − **R**^**C**^_*i*_) and for IVA **E**^**V**^_*i*_ = *abs* (**R**^**G**^_*i*_ − **R**^**V**^_*i*_). We define **E**^**C**^ as the mean of **E**^**C**^_*i*_ across all subjects, similarly **E**^**V**^ for IVA. To get a better handle on the performance of the algorithms, we report the averages and standard deviations of the diagonal and the non-diagonal elements of the error matrices separately. Let μ^*d*,*C*^_*i*_ = *mean* (*diagonal* (**E**^*C*^_*i*_)) and μ^*n*,*C*^_*i*_ = *mean* (*nondiagonal* (**E**^**C**^_*i*_)) be the *ith* subject's mean value of the diagonal and non-diagonal elements of **E**^**C**^_*i*_ and for **E**^**V**^_*i*_ as μ^*d*,*V*^_*i*_ and μ^*n*,*V*^_*i*_. Let the standard deviations of correlation errors be σ^*d*,*C*^_*i*_, σ^*n*,*C*^_*i*_, σ^*d*,*V*^_*i*_, and σ^*n*,*V*^_*i*_. In experiments where we check correlation errors across different degrees of spatial variability (*Experiment 2–4*), we report the overall mean correlation error across all subjects and all components and this we denote by μ^*d*,*C*^_*all*_ and μ^*n*,*C*^_*all*_ for mean diagonal and non-diagonal errors, respectively, across all subjects for GICA similarly by μ^*d*,*V*^_*all*_ and μ^*n*,*V*^_*all*_ for IVA. For standard deviations the following parameters will be used: σ^*d*,*C*^_*all*_, σ^*n*,*C*^_*all*_, σ^*d*,*V*^_*all*_, and σ^*n*,*V*^_*all*_.

## Results

### Experiment 1: variation in component amplitude

The simulation results of this experiment are presented in Figure 2: where Sub1's ground truth (GND) SMs is in 1st row, GICA SMs in 2nd row and IVA SMs in 3rd row. Both GICA and IVA performed near perfect reconstruction of all the SMs. In Figure 2B, the GND TC is presented in black ink and the errors between GND and GICA in blue and GND and IVA in red. TC error for Sub1 was close to zero in both GICA and IVA. All other subjects' SMs and TCs were very similar to Sub1's maps shown in Figures 1A,B; due to space limitations we do not present them here.

Correlation between GND SM and GICA SM were higher than 0.999 for all SMs and all subjects. TC correlations between GND and GICA reconstructions were upwards of 0.999 for all component TCs and subjects. Our results indicate that SMs (and TCs) were very well estimated irrespective of variability in their amplitude.

IVA estimates of SMs and TCs were also very close to GND, but the components were less clean than the GICA estimates. As seen in Figure 2A (3rd row) the IVA SMs had minor artifacts from other components. Correlations between the GND and IVA SMs were upwards of 0.991 for all components and subjects. Correlation between GND and IVA TCs were above 0.997.

In Figure 2E, we plot the product of the standard deviation of the TC (σ_*TC*_) and the maximum intensity of SM (*max*_*SM*_) vs. the component amplitude. Both methods' estimates had generally a linear association between GND amplitudes (*Amp*_*GND*_) and estimated amplitudes (*Amp*_*EST*_).

### Experiment 2: spatial variation in one component

**Figure 3:**
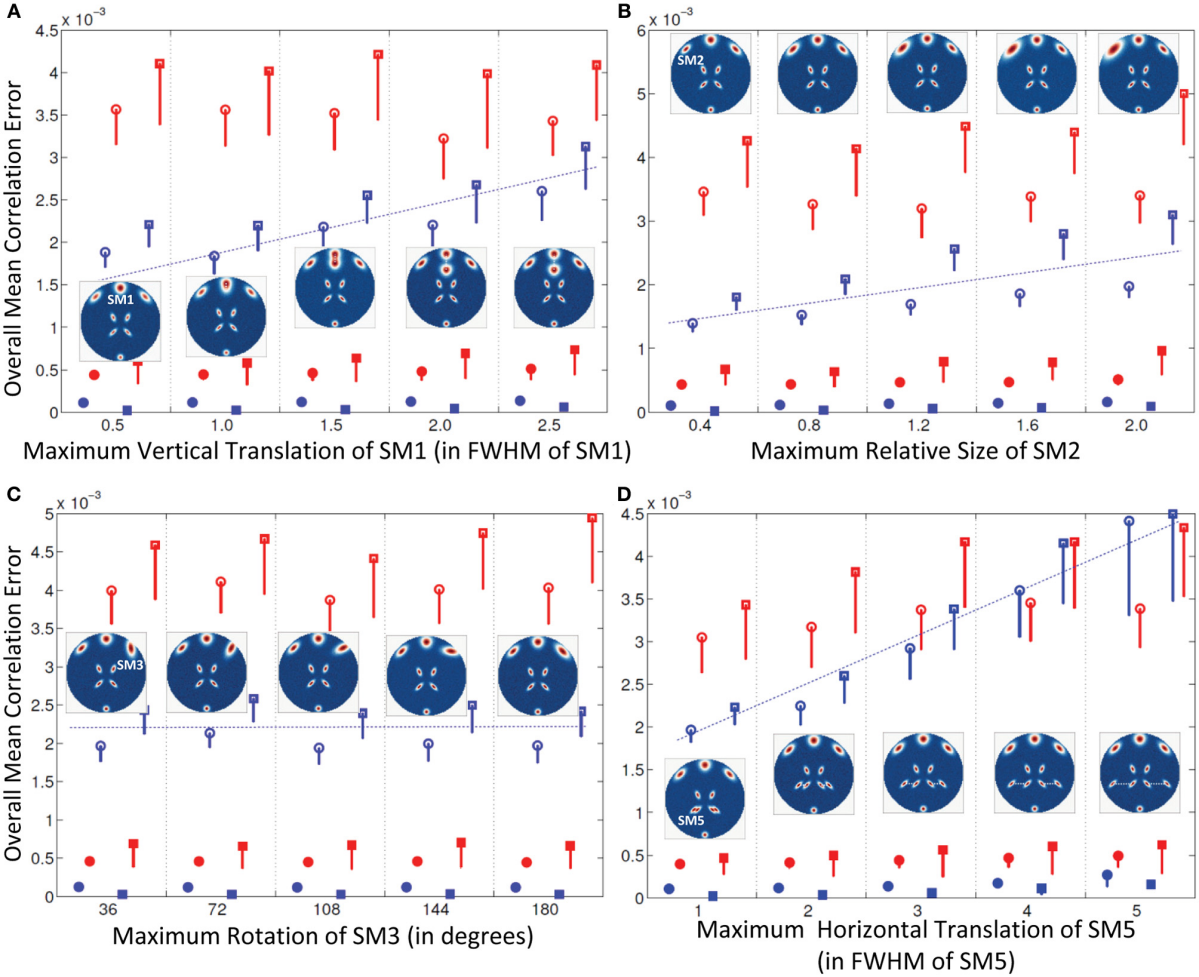
**Results of Experiment 2: Estimation accuracy when one spatial map (SM) is varied in subjects**. Errors reported here are the overall mean correlation errors (across all components and subjects); see “Definition of Result Evaluation Parameters” section for definitions. Mean errors are in blue for GICA and in red for IVA. Diagonal elements of the correlation error matrix are in filled shapes and non-diagonal elements in unfilled shapes. SMs are presented in circles and TCs in squares. *X*-axis represents the maximum level of a single SM's variability between Subjects 1 and 5; other subjects' SMs are varied uniformly between Subjects 1 and 5 as illustrated by the SM maps presented within each plot. Minimal correlation errors (<10^−3^) observed in both GICA and IVA, non-diagonal elements of GICA show an increasing trend at higher levels of variability. **(A)** Intersubject variability in the vertical translation of SM1, **(B)** Intersubject variability in the size of SM2; **(C)** Intersubject variability in the orientation (rotation) of SM3; **(D)** Intersubject variability in the horizontal translation of SM5.

Results of this experiment are presented in Figure 3. The maximum degree of spatial variation (Δ*d*_*max*_, Δ*s*_*max*_, and Δθ_*max*_) are along the *x*-axis and the mean correlation error across all subjects and components (μ^*d*,*C*^_*all*_, μ^*n*,*C*^_*all*_, μ^*d*,*V*^_*all*_, and μ^*n*,*V*^_*all*_), are along *y*-axis The error bars correspond to the standard deviation of the errors (σ^*d*,*C*^_*all*_, σ^*n*,*C*^_*all*_, σ^*d*,*V*^_*all*_, and σ^*n*,*V*^_*all*_). Only the lower bounds of the error bars are presented to provide more resolution to smaller errors in the plot.

#### Experiment 2a (vertical translation of SM1, see Figure 3A)

All mean errors were less than 4.5 × 10^−3^ for both GICA and IVA. The mean error of the non-diagonal elements for both SM and TC gradually increased in GICA (from ~2 × 10^−3^ to ~3 × 10^−3^) with increase in Δ*d*_*max*_. In IVA, although the errors were higher than GICA, we did not observe this trend of gradual increase in error with an increase in Δ*d*_*max*_. The important observation from this experiment was that in both algorithms there was no clear break down of estimation accuracy as the amount of translation increased.

#### Experiment 2b (size of SM2, see Figure 3B)

Estimates of both GICA and IVA were close to the GND (all mean errors <5 × 10^−3^). Here again, GICA errors were marginally less than IVA but there was a gradual increase in GICA non-diagonal mean correlation errors with an increase in the size variability of the component.

#### Experiment 2c (rotation of SM3, see Figure 3C)

All mean correlation errors across subjects and components were less than <5 × 10^−3^, with GICA marginally outperforming IVA. There was no increase in error with higher maximum rotation.

#### Experiment 2d (horizontal separation of SM5, see Figure 3D)

All mean errors were less than 4.5×10^−3^ for both GICA and IVA.

#### Experiment 2e (component overlap, see Figure 4)

**Figure 4:**
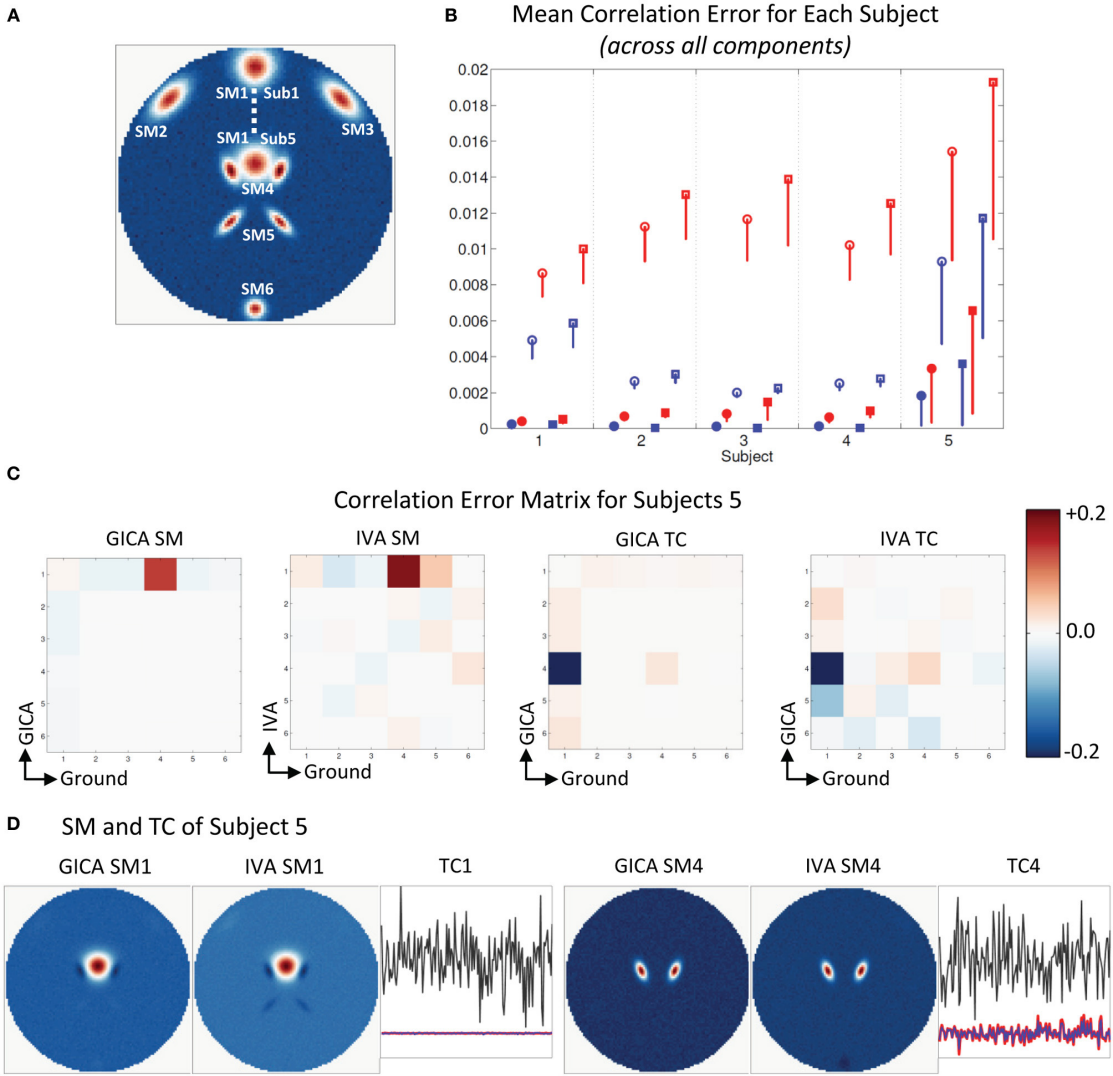
**Results of Experiment 2e: Estimation when components overlap spatially. (A)** Ground truth SMs: SM2 to SM6 for all subjects are at the same location, SM1 of Subjects 1 and 5 are presented, SM1 for Subjects 2–4 are equally distributed between locations of Subjects 1 and 5. In Subject 5, SM1 slightly overlaps with SM4. **(B)** Mean correlation errors across all components, presented for each subject separately. Mean errors are in blue for GICA and in red for IVA. Diagonal elements of the correlation error matrix are in filled shapes and non-diagonal in unfilled shapes. SMs are presented in circles and TCs in squares (see “Definition of Result Evaluation Parameters” section for their definitions): correlation errors are small in Subjects 1–4, large errors in Subject 5 where SM1 and SM4 were overlapping. **(C)** Correlation error matrix for Subject 5 indicates that both GICA and IVA have removed the spatial correlation that existed (due to overlap) between SM1 and SM4 (diagonal element) and have introduced non-diagonal correlations between SM1 and SM4 and TC4 and TC1 that did not exist in the ground truth. Applying GICA/IVA can remove spatial correlations but will introduce artificial correlations in the temporal domain. **(D)** In both GICA and IVA estimates, we observe that SM1 has negative lobes in regions of overlap with SM4 and these negative lobes are causing the zero correlations between SM1 and SM4. SM4 is well reconstructed by both methods, but TC4 has high errors and has correlations with TC1.

In Figure 4A, we see that GND SM2 to SM5 are at the same location for all subjects and that SM1 was varied in all subjects. In addition, we see that in Sub5 SM1 slightly overlaps with SM4. In Figure 4B, we present the mean correlation error for all five subjects calculated across all the components. For subjects 1–4, for both SM and TC, μ^*d*,*C*^_*i*_, and μ^*d*,*V*^_*i*_ were near zero. In Sub5, where SM1 slightly overlapped with SM4, mean correlation errors were higher. The errors of Sub5 have higher standard deviation indicating that it may be as a result of just a few elements of the correlation error matrix. Upon closer inspection of the correlation error matrix of Sub5 (Figure 4C) we see that most of the elements have lower correlation errors except errors between GND SM4 and EST SM1 and GND TC1 and EST TC4. The correlation between GND SM1 and GND SM4 in Sub5 was equal to 0.13, and this was due to their slight spatial overlap. In the estimated components correlation between SM1 and SM4 in GICA and IVA was −0.003 and 0.05, respectively. In the time domain, the correlation between GND TC1 and GND TC4 in Sub5 was near zero (= −0.002). In the estimated TCs, correlation between TC1 and TC4 was equal to 0.2 in GICA and 0.26 in IVA. In Figure 4D, we see that the near zero correlation between the EST SM1 and EST SM4 is created by SM1 having negative lobes in the overlapping region with SM4 and due to this same reason there is higher correlation between GND SM4 and EST SM1.

#### Experiment 2f (over estimation of number of components, see Figure 5)

**Figure 5:**
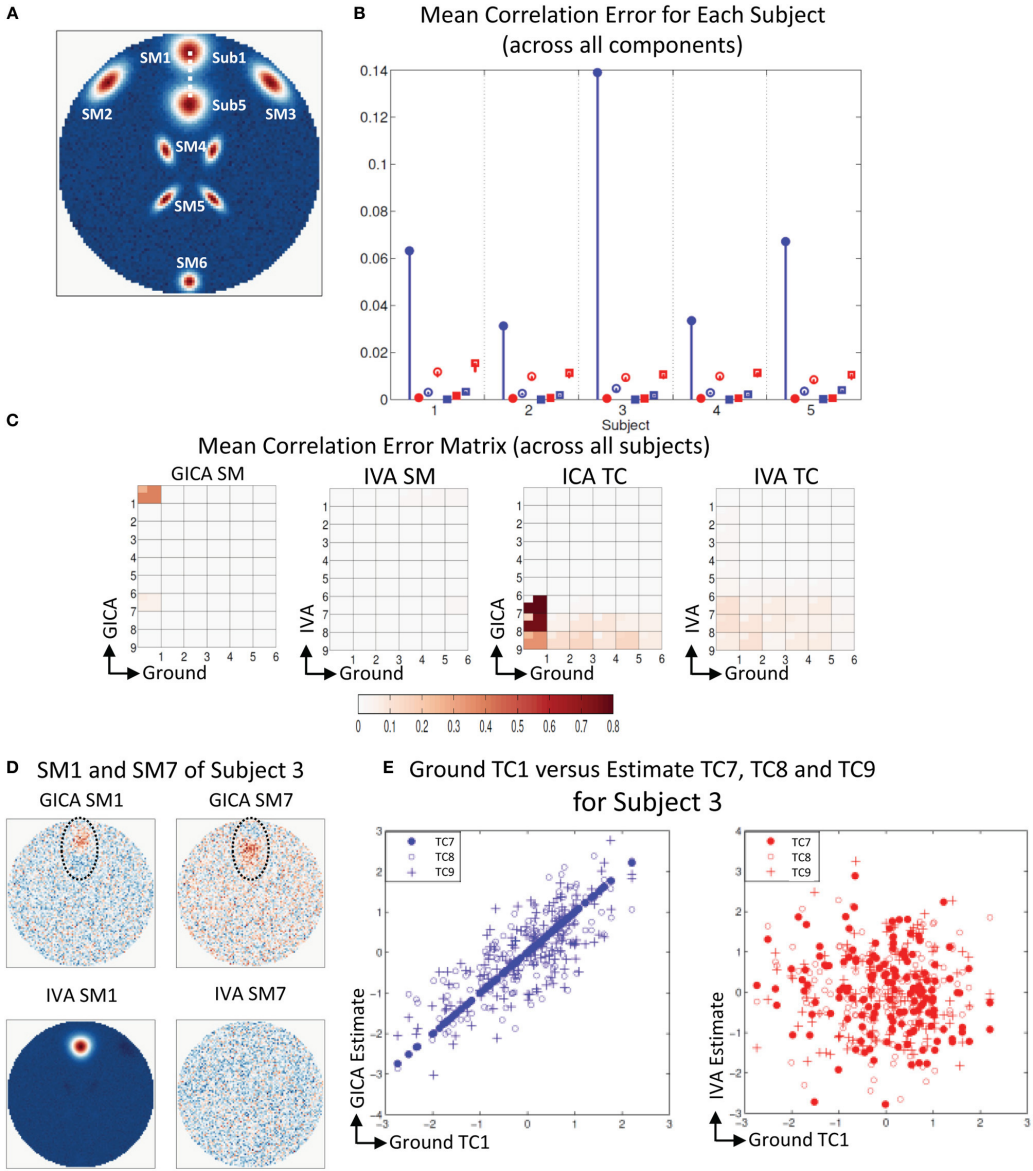
**Results of Experiment 2f: Over estimation of components (6 components are estimated with 9). (A)** All SMs, except SM1, spatially overlap across subjects. SM1 is spatially varied for all subjects. Locations of SM1 for Subjects 1 and 5 are shown and SM1 for other subjects are equally spaced between those locations. **(B)** Mean correlation errors across all components, presented for each subject separately. Mean errors are in blue for GICA and in red for IVA. Diagonal elements of the correlation error matrix are in filled shapes and non-diagonal in unfilled shapes. SMs are presented in circles and TCs in squares (see “Definition of Result Evaluation Parameters” section for their definitions). GICA diagonal SM correlation error is high with a high error bar; Subject 3 has the largest mean error. **(C)** Mean correlation error matrix between ground and estimates is calculated for all subjects and the mean and standard deviation (mini-cell inside each cell) is presented for GICA and IVA for SM and TC separately. GICA produces errors in SM1 and SM7 and large errors in TC7, TC8, and TC9. **(D)** In Subject 3, SM1 shows noisy lobes (both positive and negative) at locations where SM1 was varied between subjects. The extra component (SM7) showed similar lobe patterns. In IVA SM1 is well reconstructed and the extra component (SM7) appears as a noisy frame. **(E)** Scatter plots of the Ground TC1 vs. GICA estimates of the extra components (TC7, TC8, and TC9) show significant correlation between them. In IVA TCs of extra components are not correlated with Ground TC1.

Figure 5A shows that all SMs except SM1 has subject variability. In Figure 5B, μ^*d*,*C*^_*i*_, μ^*d*,*V*^_*i*_, μ^*n*,*C*^_*i*_, and μ^*n*,*V*^_*i*_ are presented for all five subjects. In all five subjects μ^*d*,*C*^_*i*_ was between 0.03 − 0.14 and μ^*d*,*V*^_*i*_ < 10^−3^. GICA errors had a high standard deviation, indicating that outliers may be causing the error (again, only the lower bounds of the error bars are shown in the figure to illustrate higher resolution at lower error level). The non-diagonal elements of the SM correlation matrix show marginal error in both techniques with small standard deviation. The diagonal elements of the TC correlation error matrix show very low errors in both GICA (<10^−3^) and IVA (<10^−2^). In Figure 5C, we present the mean correlation error matrices and this time the averages are computed across subjects with standard errors represented by the mini-cell within each cell. The GND-GND matrix was of size [6× 6] and due to overestimation the GND-EST correlation matrix was of size [9× 6]. The correlation error matrix indicates that the mean correlation error across subjects between GND SM1 and EST SM1 was equal to 0.4 in GICA and 0.001 in IVA. This was due to the poor reconstruction of SM1 by GICA. The worst performance of GICA was observed in Sub3 and in Figure 5D we present GICA and IVA SM1 and SM7 of Sub3. GICA SM1 and GND SM1 of Sub3 had a correlation of 0.16 and the same in IVA was 0.99. In both GICA and IVA the correlations between the extra components (SM7 to SM9), after the best pairs were matched, were all less than 0.006, except the correlation between GICA SM7 and GND SM1. In Sub3, GICA SM7 had a correlation of 0.16 with GND SM1. Overestimation in GICA essentially splits SM1, the component that was varied across subjects, into more than one estimate of SM1. The TCs of the extra GICA components showed significant correlation errors with GND TC1. The mean correlation between GICA TC7, TC8, and TC9 and GND TC1 were 0.999, 0.76, and 0.34 respectively. In IVA the same values were 0.103, 0.109, and 0.07. In Figure 5E we present a scatter plot of GND TC1 with GICA and IVA TC7, TC8, and TC9. It is clear that in GICA all TCs of the extra components have much higher correlation with GND TC1 than the extra components of IVA.

#### Experiment 2g (missing components)

In the first part of this experiment, where one SM was missing in just one subject, IVA reconstructed the SMs and TCs very close to the ground truth (correlation errors less than 0.06). In GICA SMs were closer to the ground truth but the TCs showed large errors. The TC of the component corresponding to the component that was not present in Sub1 showed high correlations errors (0.2–0.8). This was observed only in Sub1 and all other subject TCs were more accurately reconstructed in GICA. In the second part of the experiment where SM1 was present in Sub1 and missing in all other subjects, IVA continued to accurately estimate both SMs and TCs. GICA SM estimates were close to ground truth, but TC estimates of the missing component had high correlation errors with other components in some of the subjects.

### Experiment 3: spatial variation in two components

#### Experiment 3a (vertical translation of SM1 and size change of SM2, see Figure 6A)

**Figure 6:**
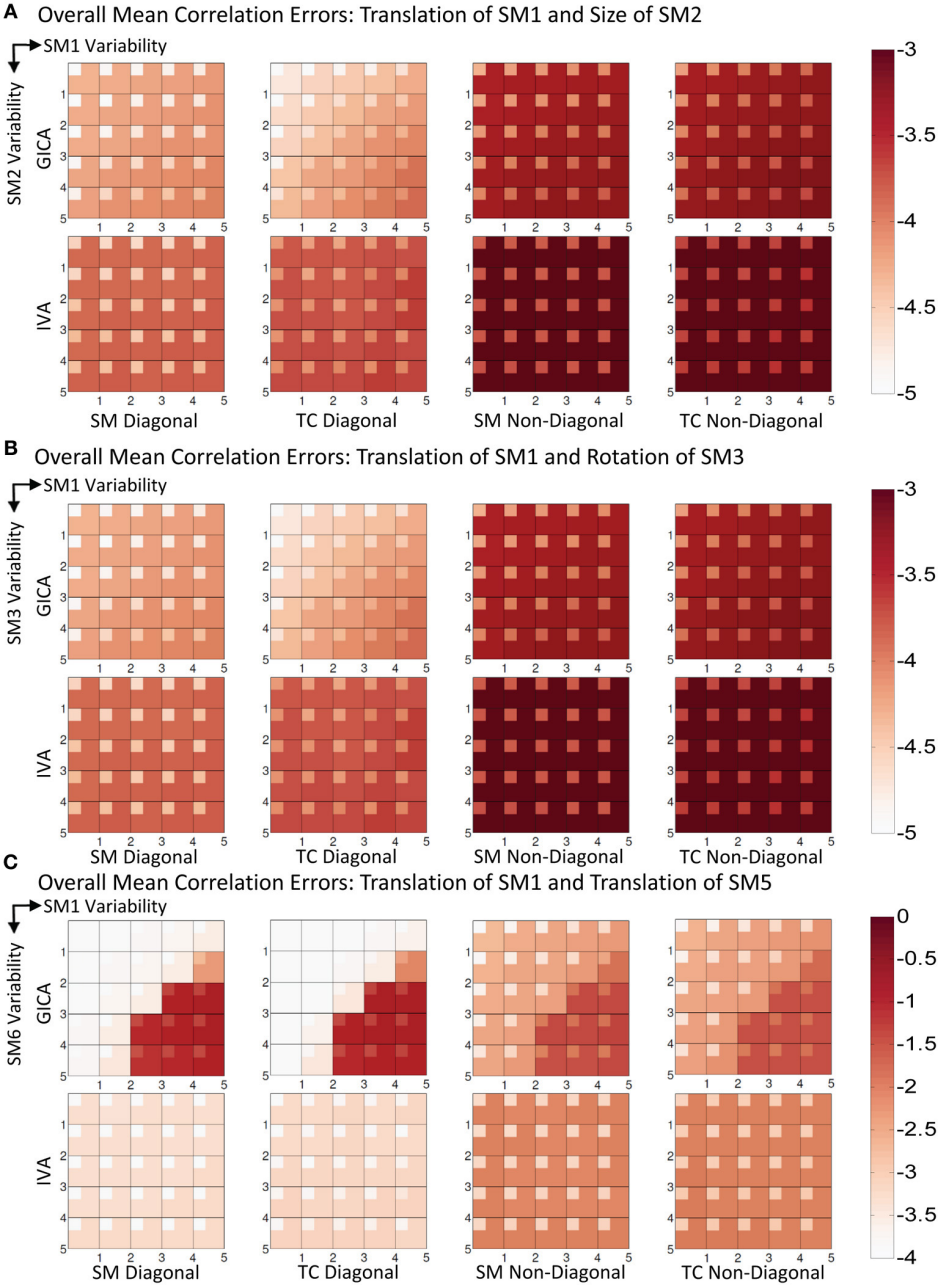
**Results of Experiment 3: Estimation when two components are varied across subjects**. Overall mean correlation errors (across all subjects and components) are presented as images for diagonal and non-diagonal elements of for SM and TC separately. Increase in variability is from left to right of the image and from top to bottom. For details on the degree of variability at each step refer to the “Simulation Experiment Setup, Experiment 3” section. The errors are in log10 scale as indicated by the colorbar. **(A)** Translation of SM1 and size change of SM2. Both GICA and IVA errors are small, In GICA, errors are marginally increasing in the diagonal direction of the image. **(B)** Translation of SM1 and Rotation of SM3. Both GICA and IVA errors are small. In GICA errors are marginally increasing in the diagonal direction of the image. **(C)** Translation of SM1 and Translation of SM5. In IVA, errors still remain small across all levels of variability. In GICA, errors are small up to the third step, but thereafter give large errors. Note the change of colorbar values for this result, indicating higher levels of correlation errors.

In Figure 6A, we present the mean correlation errors across all subjects and components (μ^*d*,*C*^_*all*_, μ^*d*,*V*^_*all*_, μ^*n*,*C*^_*all*_, and μ^*n*,*V*^_*all*_) as images for each value of Δ*d*_*max*_ and Δ*s*_*max*_. The rows in Figure 6A, from top to bottom, represent increasing degrees of vertical translation of SM1 and columns, from left to right, represent increasing degrees of size change in SM2. Both GICA and IVA do an excellent job in component estimation. In Figure 6A, we present mean errors in diagonal SM, diagonal TC, non-diagonal SM, and non-diagonal TC. All mean correlation errors were less than 10^−3^ for both GICA and IVA. GICA errors were slightly less that ICA but the errors marginally increased in the diagonal direction, that is, errors were higher at higher variability.

#### Experiment 3b (vertical translation of SM1 and rotation of SM3, see Figure 6B)

Results were similar to that of the Experiment 3a.

#### Experiment 3c (vertical translation of SM1 and horizontal separation of SM5, see Figures 6C, 7)

Results indicate much better estimation accuracy in IVA. The mean values of the diagonal elements of the SM error correlation matrix indicate that up to Δ*d*_*max*_ translation of 1.5 FWHM of SM1 and 3 FWHM of SM5 GICA estimates are accurate. After this threshold there is a sudden increase in correlation error in the range of 0.1–0.2. For IVA, μ^*d*,*V*^_*all*_ for both SM and TC correlation errors were less than 10^−3^, and there was no clear jump in error with increasing level of variability.

**Figure 7:**
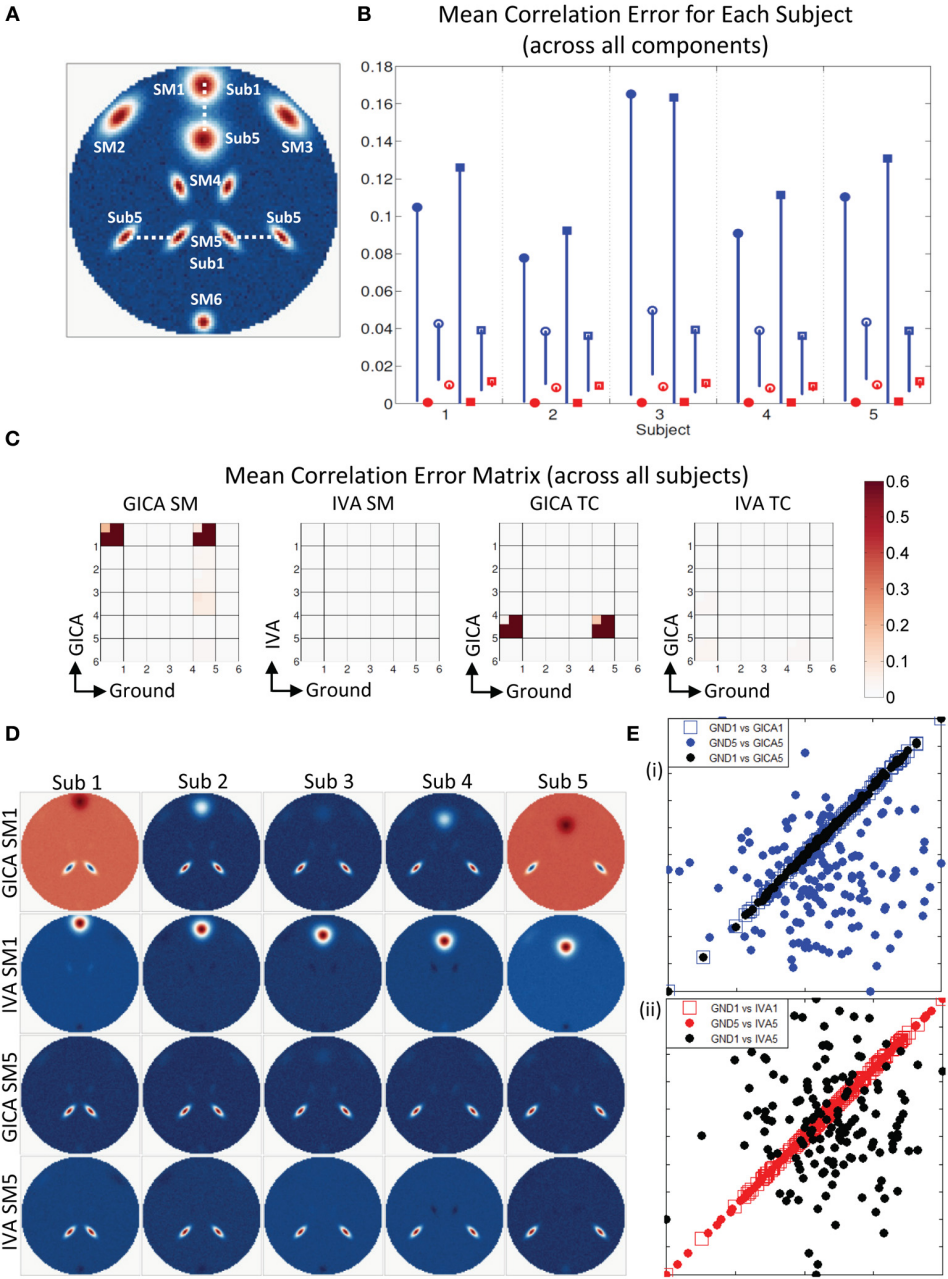
**Estimation when SM1 and SM5 are varied across subjects. (A)** SM1 and SM5 are spatially varied for all subjects. Locations of SM1 for Subjects 1 and 5 are shown and SM1 for other subjects are equally spaced between those locations. **(B)** Mean correlation errors across all components, presented for each subject separately. Mean errors are in blue for GICA and in red for IVA. Diagonal elements of the correlation error matrix are in filled shapes and non-diagonal in unfilled shapes. SMs are presented in circles and TCs in squares (see “Definition of Result Evaluation Parameters” section for their definitions). All IVA mean correlation errors are less than 0.02. All GICA errors are much higher with large error bars. For clarity of lower error values, only the lower bounds of the error bar are presented. **(C)** Mean correlation error matrix between ground and estimates is calculated for all subjects and the mean and standard deviation (mini-cell inside each cell) is presented for SM and TC separately. GICA produces large errors in the components (SM1 and SM5) that were varied across subjects. **(D)** SM1 and SM5 across all subjects. IVA produces estimates SMs with little artifact. GICA SM1 has large artifacts from SM4 and the blob of SM1 appears weak; in Subject 3 it is hardly visible. GICA estimates of SM5 are close to ground truth. **(E)** Scatter plots of the Ground TC1 vs. GICA TC1 is in filled blue circle, Ground TC5 vs. GICA TC5 is in unfilled blue square and Ground TC1 vs. GICA TC5 in filled black circles; same for IVA are in red in the next plot. GICA TC1 is highly correlated with Ground TC1, but GICA TC5 is not correlated with Ground TC5 but has high correlation with ground TC1. GICA incorrectly assigns the TC of one spatially varying SM to another spatially varying SM. In IVA TC correlations are correctly estimated.

In Figure 7B, we display the mean errors for each subject separately at SM1 Δ*d*_*max*_ = 20 voxels and SM5 Δ*d*_*max*_ = 20 voxels. This level of variability was selected as a representative example to further examine the nature of errors. The mean errors in SM and TC were much higher in GICA than IVA for diagonal and non-diagonal elements of the correlation error matrix.

Motivated by the large error bars, we further investigated the mean correlation error matrices (mean values were calculated across subjects). The mean correlation error images across subjects are presented in Figure 7C. In GICA there are large errors in the components that varied across subjects (Component 1 and 5) in both SM and TC. In IVA, all correlation errors across all subjects were less than 0.04. Mean correlation errors in TCs were high in GICA between GND TC5 and GICA TC5. Correlation error was also high in GICA between GND TC1 and GICA TC5. All TC correlation errors in IVA were less than 0.05.

In Figures 7D,E, we present the SMs and scatter plots of the TCs of components 1 and 5. Our first observation is that IVA SMs were well reconstructed with only minor artifacts. GICA SMs show extreme errors in SM1. In all Subjects the main lobe of SM1 appears less prominently than the artifact from SM5. In Sub3 the main blob of SM1 is hardly visible. SM5 in GICA was well estimated for all subjects with minor artifacts, but TC5 in GICA shows large errors. In Figure 7E (i), we present Sub3 scatter plots of the GND TC1 vs. GICA TC1 in blue filled circles, GND TC5 vs. GICA TC5 in blue unfilled circles and GND TC1 vs. GICA TC5 in black un-filled circles. Similar plots are presented for IVA in Figure 7E (ii). It is evident that GND TC1 and GICA TC1 have near perfect correlation, but GND TC5 and GICA TC5 have very weaker correlation. GICA TC5 has a much higher correlation with GND TC1. Sub3 had the poorest estimation of SM1 (correlation error = 0.96) and the poorest estimation of TC5 (correlation error = 0.96). In addition in Sub3 GICA SM1 had a high correlation with GND SM5 (correlation error = 0.98) and GICA TC5 had a high correlation with GND TC1 (correlation error = 0.98). IVA TCs were well reconstructed with appropriate correlations.

### Experiment 4: spatial variability in all components

#### Experiment 4a (uniform distribution of variability, see Figure 8A)

**Figure 8:**
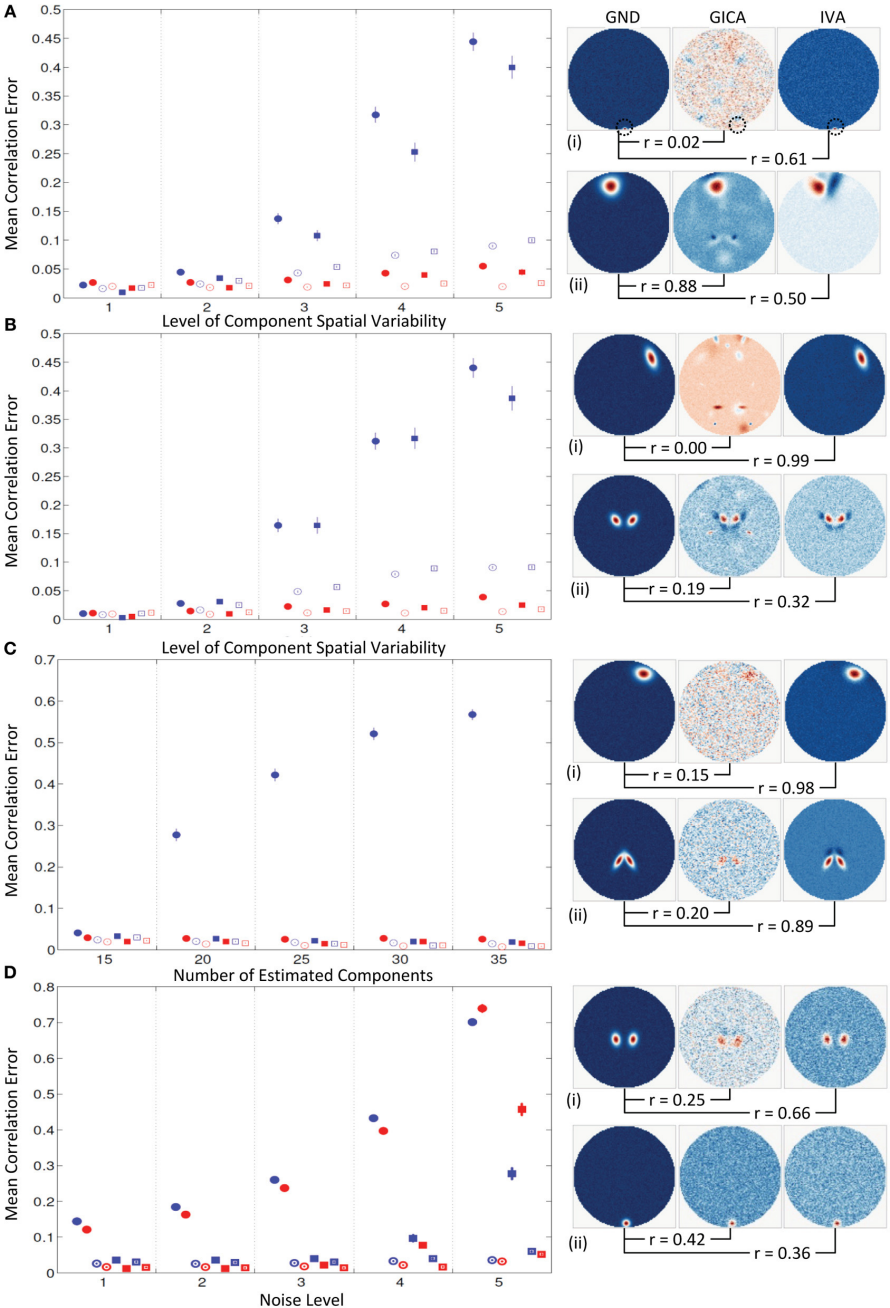
**Results of Experiment 4: Results from a larger dataset with higher number of subjects and components and with variability in all components**. In this experiment all components of all subjects undergo a combination of variability. Correlation errors reported here are mean values across all subjects and components. Mean errors are in blue for GICA and in red for IVA. Diagonal elements of the correlation error matrix are in filled shapes and non-diagonal elements in unfilled shapes. SMs are presented in circles and TCs in squares (see “Definition of Result Evaluation Parameters” section for their definitions). Next to each error plot, are SMs with least correlation with ground, for GICA in (i), and IVA in (ii). These SMs are presented along with the corresponding SM from the other technique. **(A)** When variability is drawn from a uniform distribution. GICA and IVA errors are comparable at lower variability, but GICA errors increase with increase in the level of variability. **(B)** When variability is drawn from a Gaussian distribution; results are similar to that of uniform distribution. **(C)** When variability is drawn from mid-level variability, but the number of components is overestimated. At 15 components (actual number of components as in ground truth) GICA and IVA errors are comparable. With increase in number of estimated components, GICA SM diagonal errors increase, whereas IVA errors continue to be constant. **(D)** Performance with increase in noise level.

Mean errors across all subjects and components at increasing levels of variability (along x-axis). At *k* = 1, all mean correlation errors in both GICA and IVA were less than 0.02. At *k* = 2, GICA errors begin to become marginally higher than IVA. At *k* = 3, GICA diagonal errors begin to show much higher errors than IVA in both SM and TC components. Non-diagonal elements of the correlation error matrices are also higher in GICA than in IVA. At *k* = 4 and 5 GICA diagonal correlation errors increase rapidly compared to errors in IVA. In Figure 8A (ii), we present the SM with the highest correlation error of GICA along with the corresponding GND and IVA SMs. Similarly, in Figure 8A (iii) we present the SM with the highest correlation error of IVA along with the corresponding GND and GICA SMs. Correlations of these SMs between the GND and estimates are presented below the SMs.

#### Experiment 4b (normal distribution of variability, see Figure 8B)

Results of this experiment were very similar to results of *Experiment 4a.*

#### Experiment 4c (model order over estimation, see Figure 8C)

Mean correlation errors are presented in the y-axis and the number of estimated components are in the x-axis while the amount of spatial variability was kept at *k* = 2. At *C* = 15, the case where the number of components in the GND and the number of estimated components are equal, the performance of both methods were very similar. At *C* = 20 GICA SM diagonal errors begin to increase with. GICA SM correlation errors in the diagonal element continue to increase with higher number of estimated components. At *C* = 25, 30, and 35 μ^*d*,*C*^_*all*_ increased to 0.42, 0.52, and 0.57 respectively.

#### Experiment 4d (estimation at high noise level, see Figure 8D)

For this experiment we kept the degree of spatial variability at *k* = 2 and gradually increased the noise level. μ^*d*,*C*^_*all*_ and μ^*d*,*V*^_*all*_ increased with decrease in *CNR*.

## Discussion

In this simulation study, we evaluated the accuracy of component estimations of group independent component analysis (GICA) and independent vector analysis (IVA) under varying degrees of inter-subject spatial variability of components. By using a simulation toolbox (SimTB) to create sample fMRI data sets and a thorough set of experiments we were able to identify several interesting properties of GICA and IVA. We systematically changed the degree of subject variability and evaluated the performance of component estimation not only by measuring the changes in components that underwent spatial variability but also how this variability affected all other components' spatial maps (SMs) and time courses (TCs).

### GICA vs. IVA: methodological differences

As both GICA and IVA are extensions of ICA, inherent assumptions and limitations of ICA are common to both methods, but there are several methodological differences between GICA and IVA. The initial input data (**Y**) for both approaches are identical, but the **X** matrix in GICA and IVA algorithms is not the same. In GICA there are two levels of PCA reductions before arriving at **X**; first the subject level PCA and then the group level PCA. In IVA only a subject level PCA is performed. In GICA the group level PCA ensures that the common variances at the group level are well captured. Simulations from this study provide evidence that GICA can reconstruct individual subject variability but only up to a certain limit of subject variability. GICA maximizes the spatial independence of the components at the group level as it is applied on the two dimensional group level **X** matrix. IVA jointly maximizes two objectives on the three dimensional **X** matrix: (1) The spatial independence of within subject components and (2) dependence of “similar” components across subjects, by modeling the dependence structure of similar components.

IVA results are not limited or dependent upon the back-reconstruction methodology as back-reconstruction is not needed in IVA. In IVA, subject specific mixing coefficient matrices are kept separate from each other and the subject specific SMs and TCs are obtained by directly projecting them to their respective data. The initial SM outputs of GICA are representative of the whole group and extra steps are needed to construct the subject specific SMs and TCs using one of the back reconstruction methods. Based on the back-reconstruction methodology applied, the SMs and TCs can vary slightly, see Erhardt et al. (2011) for details.

In general, IVA seeks a decomposition of the multi-subject fMRI data to estimate sources that are independent within each dataset while also aligning the estimated sources (SMs) across subjects to maximize the dependency between the aligned sources. Thus, we can imagine an overlay of the same source (SM) for multiple subjects, where the activation regions within each form a chain of slightly overlapped regions. For GICA, recovering such a source will be difficult because the averaging power will be diminished, while IVA can exploit the overlapping regions by maximizing dependency while preserving the subject variability.

### Interpretation of results and implications

In order to better understand how inter-subject component spatial variability affected estimation accuracy, our study was broken into experiments with increasing complexity.

#### Estimations under variability in amplitude

Results indicate that both GICA and IVA approaches do an excellent estimation of both SM and TC components. Near zero elements in the cross-correlation error matrices (in both **E**^**C**^_*i*_ and **E**^**V**^_*i*_, for *i* = 1 to 5) indicate that correlations between both corresponding and other components were well estimated. Results of this experiment indicate that GICA estimates are marginally better than IVA, *max* [*diag* ((**E**^**C**^_*i*_ − **E**^**V**^_*i*_))] < 10^−3^ (see Figure 2E). From a visual inspection of GICA and IVA SMs (see Figure 2A) it is evident that GICA SMs are slightly cleaner than IVA.

#### Spatial variability in one component

We investigated the effect of inter-subject variability of three types of spatial variability: component translation, component size and component rotation. One contrasting difference between GICA and IVA estimates is that, in IVA there was no clear indication of correlation errors increasing with the increase of variability. Whereas in GICA, increase in the degree of vertical and horizontal translations and size of the component resulted in an increase in correlation errors, as indicated by the positively sloped blue line in Figure 3.

#### Spatial overlap of components

We investigated the estimation accuracy while GND SMs were slightly overlapped. Our results indicate that both GICA and IVA spatially separate the overlapped components. By “spatially separate” we mean that the spatial correlation that existed between overlapping components was not present (zero correlation) in the estimated components. The estimated maps show negative lobes in the overlapping regions. There was no correlation between the ground TCs of the overlapping components, but the estimated TCs have higher correlation. In other words, both GICA and IVA remove or nullify the spatial correlation between SMs but introduce artificial correlation in the TCs of the same components (see Figure 4C). This fault in both algorithms needs to be carefully taken into consideration when the estimated components have high spatial overlap. *Experiment 2e* was a simple experiment that showed how slight spatial overlap of just two SMs in just one subject can cause errors in both SMs and TCs of that subject. In a real fMRI data application there may be multiple spatial intersections between the estimated SMs. Careful attention is needed in such cases, especially when performing functional network connectivity (FNC) analyses, as our simulation results indicate that spatial overlap between SMs can introduce non-existent artificial correlations in TCs.

#### Model order overestimation

One of the inherent issues of blind source separation techniques such as ICA and IVA is the absence of *a priori* knowledge of the actual number of independent sources (or SMs) present in the data. An approach that has gained popularity in the recent past is to estimate a large number of components. In *Experiment 2f* we estimated nine components on a dataset that had six original components. IVA produced SMs and TCs with very low correlation errors and the extra estimates (SM7 to SM9) were noise-like and were not correlated with the original GND components. GICA SM of the component with inter-subject variability had poorer correlation with the GND SM and had artifacts (negative lobes) at spatial locations of the SM in other subjects (see Figure 5D). Further the extra SMs in the estimates showed correlations with the original GND SM that had inter-subject variability. The extra TC estimates (TC7 to TC9) had significant correlations with the TC of the component that had spatial variability across subjects. The introduction of artificial TC correlations by GICA can pose potential inaccuracies in studies where FNCs are evaluated. Our results indicate that this problem is not present in IVA as the extra SMs look noise like, meaning no clear lobes of activation and the extra TCs do not have correlations with the varying component.

#### Spatial variability in two components

Our results of *Experiment 3* indicate that GICA performs well under combinations of translational, spatial and size variability. GICA performs well when both components undergo translational variability up to approximately around 1.5–3 FWHM of the component, but GICA fails beyond that maximum spatial variability. IVA performs significantly better than GICA at higher variability. GICA SM estimates resulted as a mixture of varying components (Figure 7D). GICA also incorrectly assigned the TC of one of the spatially varying SM to the other spatially varying SM (Figure 7E).

*Missing Components:* We checked the performance of GICA and IVA when components were completely missing from one or many subjects. Here again IVA continued perform well in both spatial and time domains of the estimations. GICA did well in estimating the SMs but the TCs of the missing components were not estimated accurately.

#### Spatial variability in all components

Both GICA and IVA performed well up to variability of *k* = 2 (component translations between −4 and +4 voxels, rotations between −20° and +20° and size between 0.6 and 1.4). At inter-subject variability greater than this simulated threshold GICA begins to show large errors for both uniform and Gaussian distributions. We expected that the errors of the Gaussian distribution would be less than that for uniform distribution as more subjects are centered at zero variability in a Gaussian distribution. Results indicate that GICA errors in both cases were similar (Figures 8A,B). In Figure 8A, we present the GICA SM with the largest error. The small activation or blob (indicated by green circle) appears very noisy with many other similar size artifacts from other components in GICA. IVA identifies this component distinctly in spite of its minute size. In general, at higher levels of spatial variability, GICA SMs were a combination of many GND components. In *Experiment 4c* we performed component overestimation and results show that when the number of components is higher than the number of components in the GND, GICA produces large errors in SMs. This experiment also showed that GICA components were noisy (see Figure 8C) and the extra components had remnants of activation from other GND components. IVA recovered the SMs and TCs well and the extra components appeared noisy (an ideal result).

### GICA or IVA

Both methods yield accurate component estimations at low levels of subject variability. Prior knowledge of the degree of subject variability in the fMRI data can help to decide which method to choose. A possible approach to find the degree of component variability is to apply individual ICA on real fMRI data for a few subjects, reconstruct the components, and evaluate the spatial dissimilarities. Spatial variability across subjects may be fMRI task dependent and possibly higher in resting state data. In this study, we showed that at low levels of subject variability, GICA SMs and TCs are much cleaner than IVA. IVA does very good estimations at low levels but there were minor artifacts from other components. At higher levels of subject spatial variability of components, GICA reconstructs the components that did not have inter-subject variability well, but performs poorly on the components with subject variability. In some cases GICA estimated the SMs well but introduced errors in the TC of the component. When the number of components was overestimated in the presence of subject variability, GICA estimates of varying components and the extra components had weak activations with high levels of noise; further the TCs of the varying and extra components were correlated.

If the goal of a certain project is to obtain group level maps, then GICA is preferred to IVA. GICA components are constructed after a second level PCA reduction across the whole group. This step identifies the common patterns of activation present in the data and the constructed components represent the components corresponding to the strongest variances across the whole group. As such variances due to noise or due to minor variability in one subject are minimized in GICA. If subject specific SMs are the main interest then GICA users need to select one of the many back-reconstruction techniques available and slight differences in the SMs and TCs can be present dependent on the technique chosen.

A few impediments do exist while applying IVA as currently implemented. One constraint is the need for large memory. In our simulations we used a 64 bit laptop with 4 GB RAM and clock speed of 2.67 GHz. To estimate 5 components with 8 k voxels from 5 subjects IVA takes 1.75 s and GICA 1.3 s. To estimate data from 25 subjects with 25 components IVA took more than 16 min while GICA performed the same in less than a minute. With real fMRI brain volumes usually of size 50–100 k voxels, computer memory needed for IVA is higher than GICA by a factor of number of subjects present in the data. As such applying IVA at its current state may not be feasible with typical desktop capacities if the number of subjects, number of components and number of voxels are very large. Additional optimizations to the IVA algorithm are needed to decrease computational burden.

### Limitations and future work

#### Verification with real fMRI data

Application of IVA to real fMRI data and its performance in terms of component fidelity, implication, and robustness were not checked in this study, but through a systematic simulation framework and thorough analyses of results we provide evidence that IVA may be a better approach to capture inter-subject variability. GICA is a well-tested and widely applied technique that has provided consistent results across many different types of fMRI data (different types of task related and resting state) and across many different studies. The level of inter-subject spatial variability of components present in real fMRI data is uncertain. Further studies are needed to evaluate the performance of GICA and IVA with respect to results from individual ICA. We intend to do that in our next project.

#### Limitations of our simulation setup

The simulated datasets used possess many realistic properties but unavoidably have many limitations. It should be noted that our simulations model the fMRI data as a weighted linear mixture of spatial SMs, where the weighting is determined by TCs, in other words that the fMRI data can be given as product of SMs and TCs and adheres to the assumptions of ICA. We assigned one single TC to represent the activation changes of all the voxels of a SM. This again is an extension of ICA assumptions. Further, the TCs in our simulations were randomly generated with close to zero correlation between different components of the same subject and similar components across different subjects. The ability of both IVA and GICA to construct good SM estimates when inter-subject TC correlations for similar components are near zero is encouraging and bodes well for the algorithms. Functional network studies have indicated that higher correlations can exist between components of the same subject. In this study we did not investigate how reconstruction accuracy may change at higher correlations between TCs or the correlation limit at which ICA would begin to cluster correlated components. In addition, our datasets did not include effects of subject motion and spatial smoothing. In summary, it should be noted that real fMRI datasets are much more complex than our simple datasets. There are several back reconstruction techniques available to estimate subject specific components from group components and in our experiments we used spatio-temporal regression. We did not test other techniques for all our experiments, but in cases where there was high subject variability we checked the results with other back reconstruction techniques. Our preliminary results indicate that the errors in GICA were higher irrespective of the technique applied, but the nature of errors was different.

#### Future improvements

IVA has a high computation and memory burden compared to GICA. It should be noted that GICA has been highly optimized over many versions of the GIFT toolbox. In future work, we intend to improve these limitations of IVA. Further studies are also needed to examine if a joint framework of GICA and IVA can be developed to capture the advantages of both schemes.

### Conflict of interest statement

The authors declare that the research was conducted in the absence of any commercial or financial relationships that could be construed as a potential conflict of interest.
