# Supplementary figures and images for: Preserving subject variability in group fMRI analysis: performance evaluation of GICA vs. IVA
Source: Front Syst Neurosci. 2014 Jun 26;8:106. doi: 10.3389/fnsys.2014.00106 (PMC4071815; doi:10.3389/fnsys.2014.00106)

Supplementary Figure

Spatial Maps (total of 15) used in Experiment 4

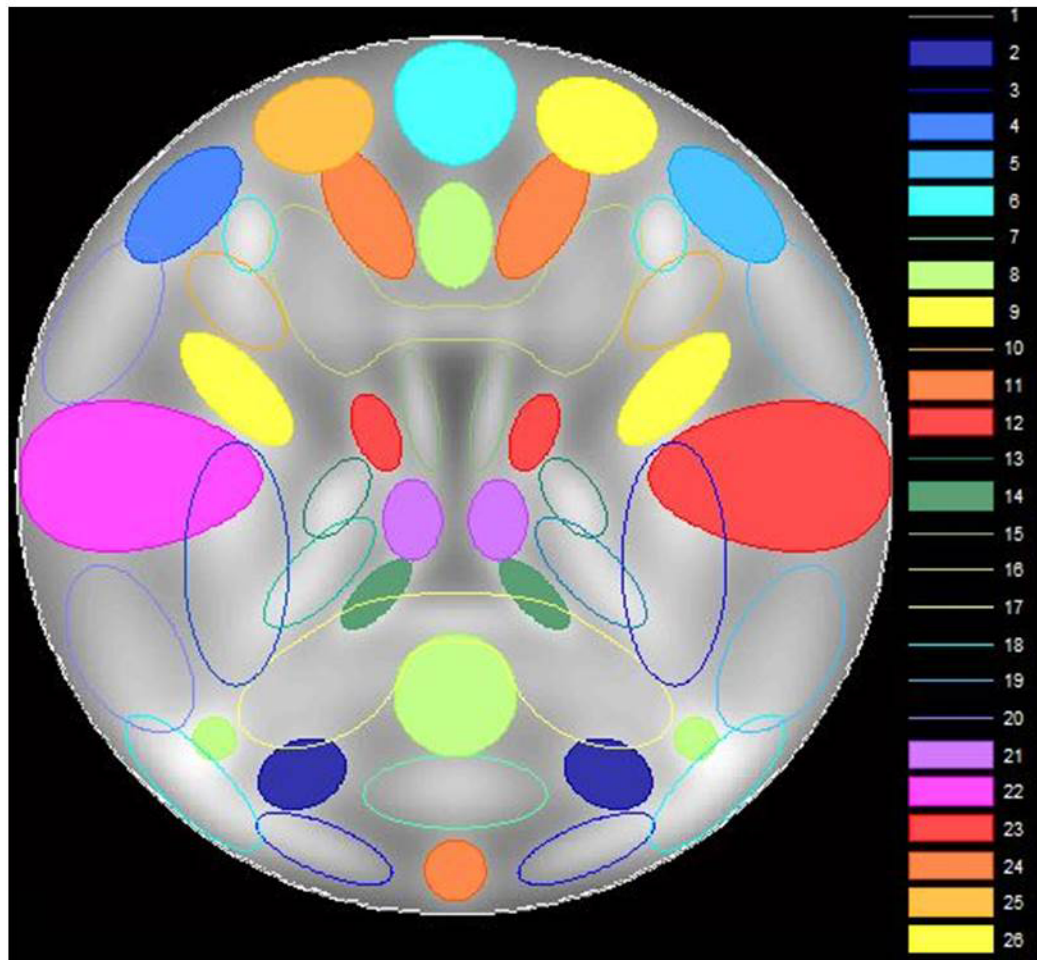

Supplement: Supplementary file 1 [file Presentation1.PDF]
